# The Art of Drawing up Special Reports

**Published:** 1913-05-10

**Authors:** 


### THE SCHOOL DOCTOR.

### III.—The Art of Drawing up Special Reports.

Medical inspectors of schools are often asked to draw up special reports on certain subjects of temporary or permanent interest. Sometimes these special subjects have a purely local interest and can hardly be said to stand in need of scientific investigation; but much more often, although especially local in interest, they are yet sufficiently important to be generally interesting. In such latter cases the school doctor would do well to pay attention to the drawing up and presenting of his report. We have before us an article on an orthopædic subject, written by a professor of world-wide reputation; in it he acknowledges his indebtedness to a provincial school doctor for an observation with regard to a certain point of more than ordinary interest. The report of that school doctor has been condensed, we believe, into a ten-line paragraph; but the labour entailed in drawing up what is probably a much longer report has not been wasted if it has attracted the attention of the professional critics.

In drawing up special reports the school doctor should bear in mind the same points that he attends to when writing an article for the professional press on a professional subject.

The mere fact that Sir Clifford Allbutt has found it worth while to compile a series of text-book notes which are even more elementary than the old-fashioned Meiklejohn with which we " stumbled across the paths of syntax " in bygone days, shows that medical men expend comparatively little trouble and patience upon their professional writing. This is much to be deplored. Interesting facts presented in an unattractive literary dress lose half their force. Statistics above all demand great care in handling. It is not everyone who has the skill and experience of Professor Pearson in presenting them to the reader in such a way that they charm instead of bore. Anyone who wishes to see for himself the difference between well-presented summaries and badly arranged tables, can find a means of comparison in studying the latest report of the Medical Officer of the Board of Education—which is an example of statistics arranged in the office—and some of Dr. Pearson's monographs, which show how craftily an able statistician, with literary bent, can charm the interest of his readers.

No special report, if it be worth the name of a report, ought to be considered complete without references. The school doctor who ventures to investigate a special subject ought to know what has been written and reported upon that subject by others, and he should give his readers references in order to enable them to look up certain points if they desire to do so. Besides, a bibliography, however partial it is, serves as a permanent guide to those who afterwards read the report, since it shows what books have been consulted. Some discrimination is to be observed in drawing up this list, and obviously only references which are really useful should be included. Thus it is not of much use to give the references to text-books which are familiar, or ought to be familiar, to every reader. On the other hand, references to out-of-the-way papers, not readily accessible, should be accompanied by a brief synopsis of the contents of these papers so far as they bear on the subject of the report.

The school doctor ought also to remember that the material he has collected for incorporation in a special report is not necessarily lost by being included in such a report. It may be used for other papers and articles, and he should keep his notes and add to them from time to time. Every doctor has his own method of note taking and preserving, and familiarity with his own method alone can render him independent and able to control the data which he has collected. Some prefer a card system, others work with notebooks, and others again prefer the method of slips; of these various methods the second is perhaps the most generally useful, especially if amplified by the use of proper record cards, such as are nowadays available under every Education Authority in whose district school doctors are at work.

Conciseness and clarity should be the primary essentials in a report, and everything that detracts from them should be relegated to the appendices.

